# TLR-2 Activation Induces Regulatory T Cells and Long-Term Suppression of Asthma Manifestations in Mice

**DOI:** 10.1371/journal.pone.0055307

**Published:** 2013-02-05

**Authors:** Martijn C. Nawijn, Alexandre C. Motta, Renée Gras, Soheila Shirinbak, Hadi Maazi, Antoon J. M. van Oosterhout

**Affiliations:** Laboratory of Allergology and Pulmonary Diseases, Department of Pathology and Medical Biology, University of Groningen, GRIAC Research Institute, University Medical Centre Groningen (UMCG), Groningen, The Netherlands; French National Centre for Scientific Research, France

## Abstract

Asthma is a chronic inflammatory disease of the airways characterized by variable airway obstruction and airway hyperresponsiveness (AHR). The T regulatory (Treg) cell subset is critically important for the regulation of immune responses. Adoptive transfer of Treg cells has been shown to be sufficient for the suppression of airway inflammation in experimental allergic asthma. Intervention strategies aimed at expanding the Treg cell population locally in the airways of sensitized individuals are therefore of high interest as a potential therapeutic treatment for allergic airway disease. Here, we aim to test whether long-term suppression of asthma manifestations can be achieved by locally expanding the Treg cell subset via intranasal administration of a TLR-2 agonist. To model therapeutic intervention aimed at expanding the endogenous Treg population in a sensitized host, we challenged OVA-sensitized mice by OVA inhalation with concomitant intranasal instillation of the TLR-2 agonist Pam3Cys, followed by an additional series of OVA challenges. Pam3Cys treatment induced an acute but transient aggravation of asthma manifestations, followed by a reduction or loss of AHR to methacholine, depending on the time between Pam3Cys treatment and OVA challenges. In addition, Pam3Cys-treatment induced significant reductions of eosinophils and increased numbers of Treg cells in the lung infiltrates. Our data show that, despite having adverse acute effects, TLR2 agonist treatment as a therapeutic intervention induces an expansion of the Treg cell population in the lungs and results in long-term protection against manifestation of allergic asthma upon subsequent allergen provocation. Our data indicate that local expansion of Tregs in allergic airway disease is an interesting therapeutic approach that warrants further investigation.

## Introduction

Allergic asthma is an inflammatory disease characterized by airway hyperresponsiveness (AHR) to bronchospasmogenic compounds, elevated allergen-specific IgE serum levels and chronic airway eosinophilia. Th2 cells and the cytokines IL-4, IL-5 and IL-13 produced by Th2 effector cells are known to be critical for the induction of allergic asthma manifestations. Hence, allergic asthma can be attributed to inadequate regulation of Th2 activity. Several studies have shown that regulatory T (Treg) cells have the ability to suppress allergic inflammation and asthma manifestations upon allergen provocation in mouse models of allergic asthma. For instance, adoptive transfer of Treg cells into allergen-sensitized mice down-regulates asthma manifestations [1], while depletion of these cells exacerbates experimental asthma [2], [3]. These data identify Treg cells as a potentially relevant target for therapeutic intervention in allergic asthma and Treg cell-based therapies are currently being considered for the treatment of this complex disease [4]. Therapeutically, expansion of the endogenous Treg cell population in allergen sensitized individuals is a more attractive approach than adoptive transfer of *ex vivo* expanded Treg cell subsets [5]. One possible approach to expand the allergen-specific Treg cell subset *in vivo* is the triggering of Toll-like receptor-2 (TLR-2) in the presence of antigen presentation [6].

Toll-like receptors are the best characterized class of pattern recognition receptors of the innate immune system, and trigger antimicrobial host defense responses [7]. TLRs recognize a wide range of molecules expressed by invading pathogens, collectively referred to as pathogen-associated molecular patterns (PAMPs). For example, TLR-4 recognizes lipopolysaccharide, a cell wall component of gram-negative bacteria. In contrast, TLR-2 recognizes a wide spectrum of PAMPs such as membrane components of Gram-positive and -negative bacterial cell wall, mycoplasma, mycobacteria, yeast and parasites [8].

TLR activation regulates the adaptive immune response by affecting DC phenotype and function [7]. Upon interaction with their ligand, TLR signaling induces DC maturation, up-regulation of co-stimulatory molecules and pro-inflammatory cytokine production [9]. In contrast, activation of certain TLRs such as TLR-7 or TLR-9 [10], [11] on plasmacytoid DCs has been shown to induce a tolerogenic phenotype. TLR activation therefore represents a crucial regulatory mechanism to mount an adequate adaptive immune response. TLRs are expressed by dendritic cells and macrophages as well as numerous other cell-types such as T cells, neutrophils, eosinophils, mast cells, monocytes and epithelial cells [7].

Several TLRs have been reported to be expressed by Treg cells and to modify their physiology [12], [13]. For instance, TLR-4 and −5 enhance the suppressive capacities of Tregs [14], [15], whereas TLR-8 activation results in suppression of Treg functions [16]. TLR-2 signaling has been shown to induce Treg cell expansion accompanied by a loss of suppressive activity *in vitro* and *in vivo* [6]. Interestingly, TLR-2 stimulated Tregs regain their *in vivo* suppressive capacity over time [17].

We hypothesized that the TLR-2-driven expansion of Tregs could be employed to increase their activity in allergen sensitized mice resulting in reduced asthma manifestations upon subsequent allergen provocations.

Here, we show that treatment of OVA sensitized mice with the TLR-2 agonist Pam3Cys induces a mild and transient exacerbation of asthma manifestations at the time of treatment. However, this effect is associated with the acquisition of increased numbers of CD4^+^CD25^+^Foxp3^+^ Treg cells in the lungs and long-term protection to asthma manifestations upon secondary allergen inhalation challenge.

## Results

### TLR-2 Triggering Induces a Trend Towards Reduced Airway Hyperresponsiveness

**Figure 1:**
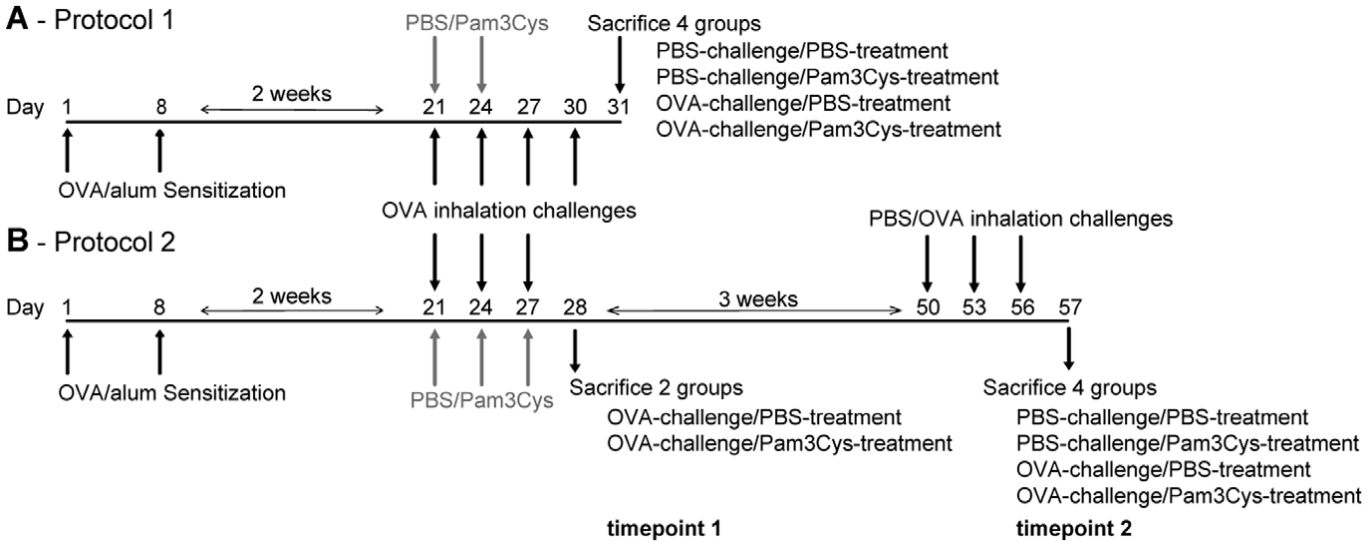
Time schedule of the experimental procedures. Days of sensitization, OVA/PBS challenges, Pam3Cys/PBS treatments and lung function measurements/section are indicated for both the short protocol (A) and the long-term protocol (B).

**Figure 2:**
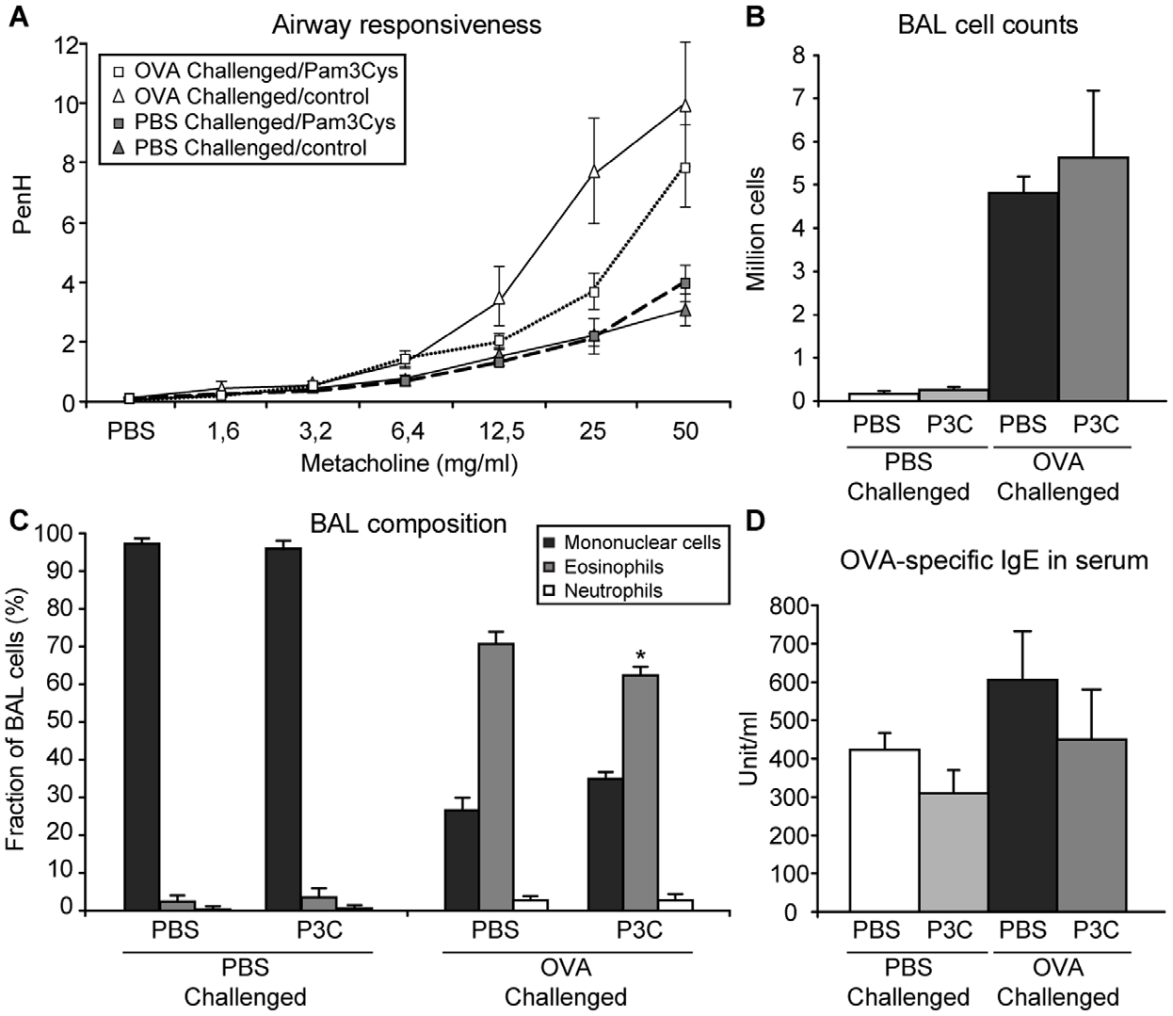
Airway responsiveness, BAL cell counts and composition, and OVA-specific IgE serum levels. Mice (n = 8) were sensitized with OVA/Alum and received 4 OVA or PBS inhalation challenges. TLR-2 agonist (20 µg per mouse in PBS) or PBS was administered intranasally 1 hour before the 2 first challenges. Asthma manifestations were measured one day after the last challenge. A: Airway responsiveness to increasing doses of methacholine was measured by whole-body plethysmography and is expressed as enhanced pause (PenH) (gray symbols: PBS-challenged groups, white symbols: OVA-challenged groups, squares: Pam3Cys-treated groups, triangles: PBS-treated groups). B, C: total (B) and differential (C) cell counts in the BAL of PBS and OVA-challenged mice as indicated. D: OVA-specific IgE levels in the serum of PBS and OVA-challenged mice as indicated. All values are displayed as average ± SEM.

It has been reported that Treg cells express TLR-2 and that TLR-2 signaling induced by Pam3Cys at the time of TCR-mediated Treg cell activation results in Treg proliferation, both *in vitro* and *in vivo* [17]. However, TLR-2 signaling is also associated with immunity enhancing effects. First, the TLR-2-induced proliferation of Treg cells is accompanied by a transient loss of their suppressive capacity [17]. Second, TLR-2 is also expressed by numerous other cell types such as macrophages, DCs and epithelial cells. In these cells TLR-2 triggering is known to induce pro-inflammatory effects such as enhanced maturation of DCs [7] and release of pro-inflammatory mediators by epithelial cells [18]. Therefore, we designed an adapted asthma protocol allowing us to circumvent the immediate pro-inflammatory effects of TLR-2 stimulation by Pam3Cys. In this protocol, OVA-sensitized mice were challenged by 4 OVA inhalations at 3 days interval. Pam3Cys or PBS as a negative control was administrated intranasally at 5 or 20 µg/mouse at the time of the 2 first challenges only (Figure 1). One day after the last challenge, mice were subjected to lung function measurements followed by section. OVA challenge in control-treated mice induced typical AHR, while mice that had received a Pam3Cys treatment at the first two OVA inhalation challenges showed a trend towards a reduced AHR (p = 0.10) (Figure 2a for the 20 µg/mouse dose of Pam3Cys). No differences were observed between the two doses of Pam3Cys tested in this experiment (data not shown).

### TLR2 Activation does not Affect Allergic Inflammation in the Lungs

To test whether the manifestations of allergic inflammation were also reduced by local Pam3Cys treatment, we analyzed OVA-specific IgE in serum and BAL cell counts. Remarkably, mice treated with Pam3Cys had comparable levels of OVA-specific IgE as PBS-treated control mice after OVA challenges (Figure 2d). Also, the total numbers of cells in BAL were not affected by the Pam3Cys treatment (Figure 2b). However, differential cell counts showed that the proportion of eosinophils was slightly but significantly decreased, while the fraction of mononuclear cells was increased in the BAL of Pam3Cys-treated mice (Figure 2c). Given the slight, not-significant increase and substantial variance in total BAL cell numbers in the Pam3Cys treatment group, this difference in eosinophil number is only observed when analyzed as a fraction of BAL cells, not when analyzed as total numbers of eosinophils in BAL. Of note, the results with regard to eosinophil cell suppression (as fraction of cells only) were identical with the two doses of Pam3Cys used (data not shown). Taken together, these data indicate that local Pam3Cys instillation at the first two out of four OVA inhalation challenges has limited effects on both eosinophilic airway inflammation and hyperresponsiveness.

### Long-term Protection Against AHR by TLR-2 Triggering

To disentangle the acute effects of Pam3Cys administration from the long-term effects, and to test whether our strategy could provide long-term protection against AHR or airway eosinophilia, we designed a new treatment protocol (Figure 1). In this protocol, 2 series of OVA challenges were performed in OVA sensitized mice with a 3 week resting period in between to allow the inflammation induced by the first series of challenges to resolve. Pam3Cys was administered exclusively during the 1^st^ series of challenges (Figure 1). To evaluate the immediate and long-term effects of TLR-2 activation, AHR was measured after both series of OVA challenges.

**Figure 3:**
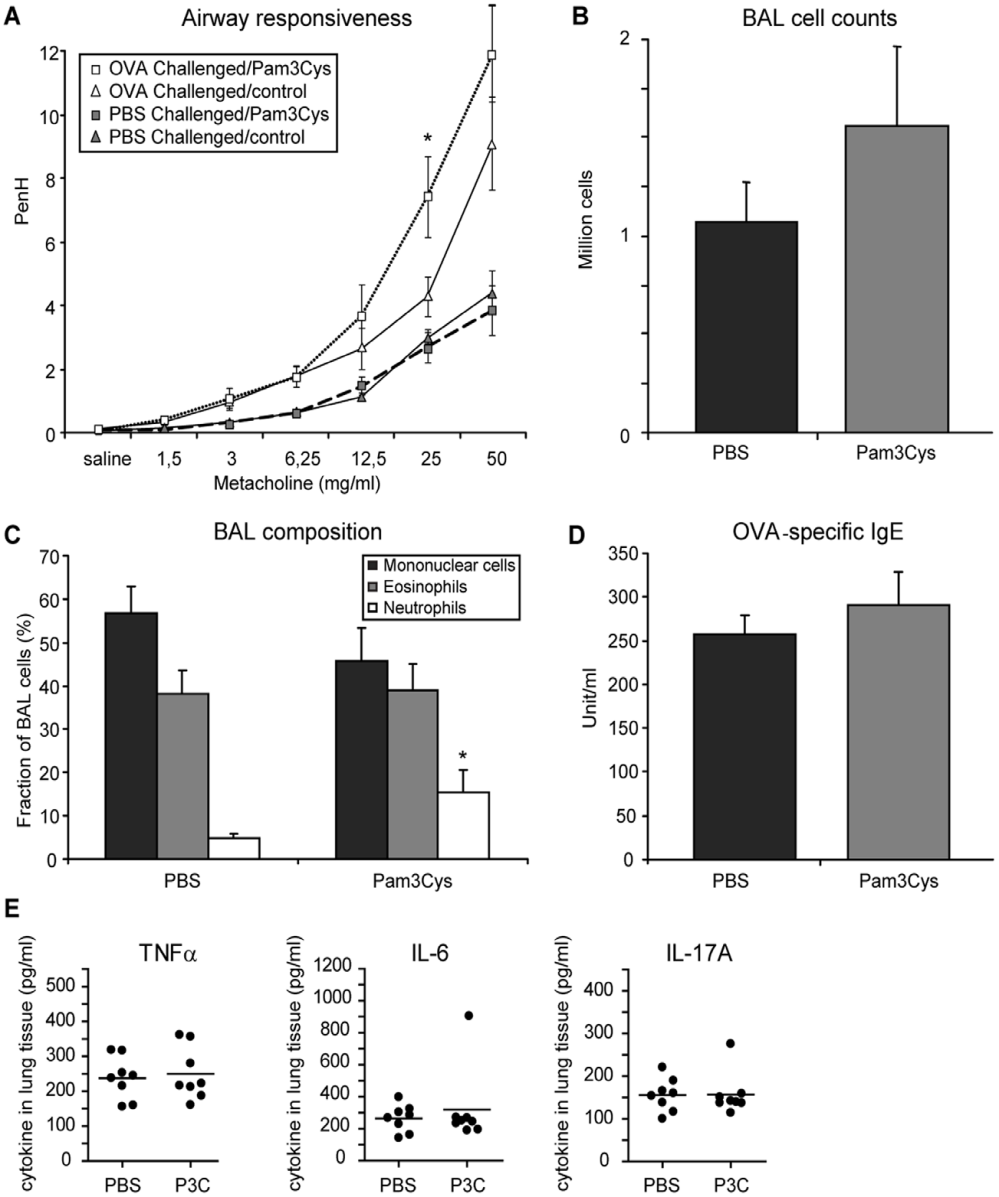
Airway responsiveness, lung cell infiltration and OVA-specific IgE serum levels. Mice (n = 8) were sensitized with OVA/Alum and received 3 OVA or PBS inhalation challenges. TLR-2 agonist (20 µg per mouse in PBS) or PBS was administered intranasally 1 hour before each challenge. Asthma manifestations were measured one day after the last challenge. A: Airway responsiveness to increasing doses of methacholine was measured by whole-body plethysmography and is expressed as enhanced pause (PenH) (gray symbols: PBS-challenged groups, white symbols: OVA-challenged groups, squares: Pam3Cys-treated groups, triangles: PBS-treated groups). B, C: total (B) and differential (C) cell counts in the BAL of OVA-challenged mice. D: OVA-specific IgE levels in the serum of OVA-challenged mice. (E) Lung tissue cytokines measured in lung homogenates of PBS or Pam3Cys (P3C) treated mice as indicated. All values are displayed as average ± SEM (panels A–D) or individual values (dots)+mean (bar) (panel E). Significance is indicated (*) when p<0.05.

**Figure 4:**
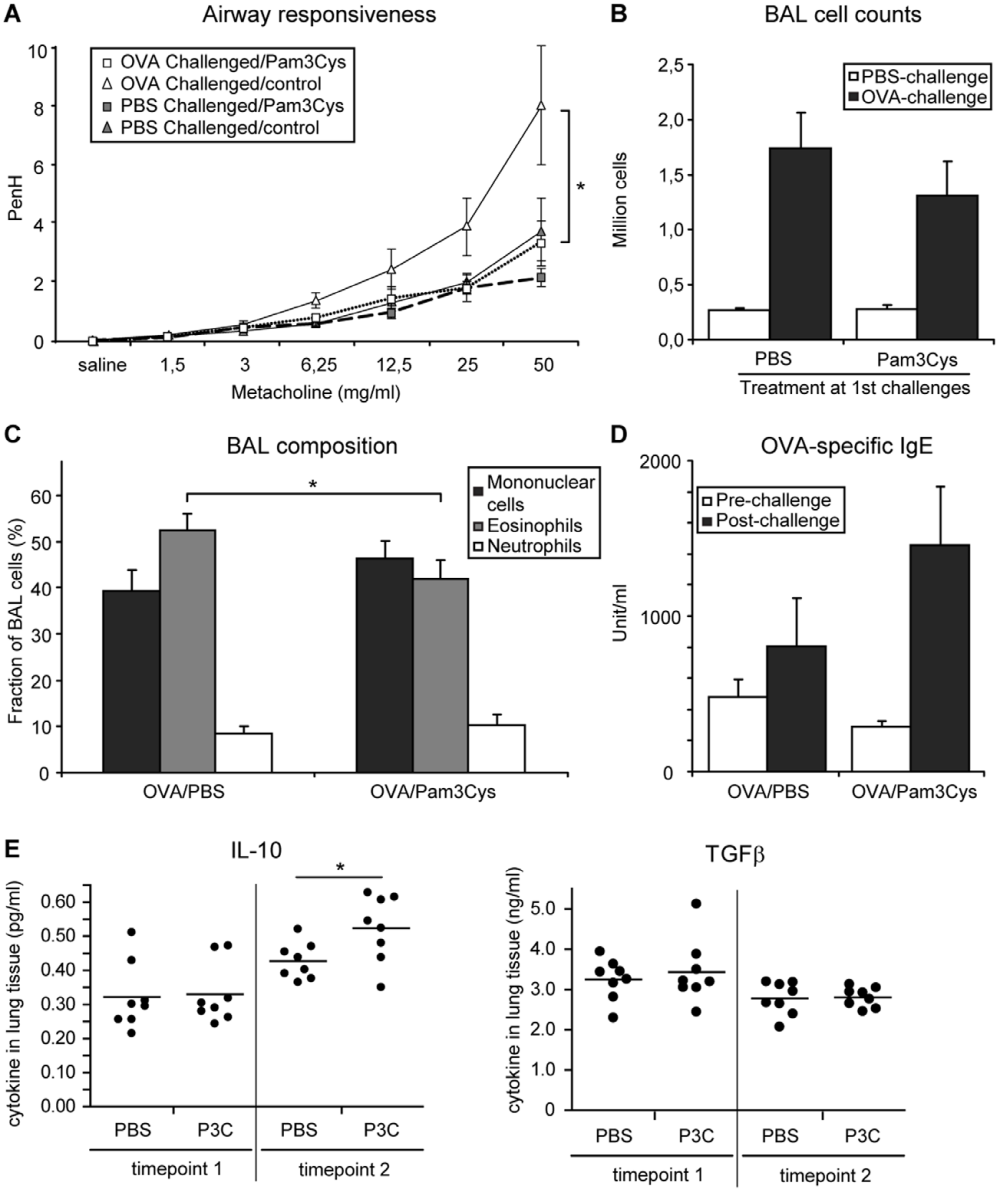
Airway responsiveness, lung cells infiltration and OVA-specific IgE serum levels. Mice (n = 8) were sensitized with OVA/Alum and received a first series of 3 OVA inhalation challenges, during which TLR-2 agonist (20 µg per mouse in PBS) or PBS was administered intranasally 1 hour before challenge. After 3 weeks, mice received a second series of 3 inhalation challenges (OVA or PBS). Asthma manifestations were measured one day after the last challenge. A: Airway responsiveness to increasing doses of methacholine was measured by whole-body plethysmography and is expressed as enhanced pause (PenH) (gray symbols: PBS-challenged groups, white symbols: OVA-challenged groups, squares: Pam3Cys-treated groups, triangles: PBS-treated groups). B: total cell counts in the BAL of PBS and OVA-challenged mice as indicated. C: differential cell counts in the BAL of OVA-challenged mice only. D: OVA-specific IgE levels in pre-challenge and post-challenge sera of OVA-challenged mice. (E) Lung tissue cytokines measured in lung homogenates of PBS or Pam3Cys (P3C) treated mice after OVA challenge at timepoint 1 (left-hand panel) or at timepoint 2 (right-hand panel) as indicated. All values in panels A–D are displayed as average ± SEM, panel E displays individual values (dots)+mean (bar). Significance is indicated (*) when p<0.05.

After the first series of challenges, mice treated with Pam3Cys displayed a moderate increase in AHR (Figure 3a). In contrast, these Pam3Cys treated mice had a complete loss of AHR at the second series of OVA challenges (in the absence of further TLR-2 activation) with overlapping responses between OVA- and PBS-challenged mice in the Pam3Cys treated group (Figure 4a).

### Long-term Effects of Pam3Cys Treatment on Allergic Inflammation

Since we had not observed a pronounced effect of Pam3Cys treatment on manifestations of allergic inflammation in our first protocol, we assessed the OVA-specific IgE and BAL cell counts in the long-term protocol both after the first and after the second series of OVA challenges. As observed in the first experiment, Pam3Cys treatment did not affect OVA-specific IgE levels in serum (Figure 3d). This was observed immediately after the Pam3Cys treatment, but also after the second series of OVA inhalation challenges (Figure 4d). In line with these data, no differences in IL-4 or IFNγ levels were observed at either timepoint in lung tissue of Pam3Cys and control-treated mice (data not shown), indicating that the Pam3Cys treatment did not induce a strong shift in the Th1/Th2 balance. To further analyze a putative pro-inflammatory activation due to the local delivery of Pam3Cys to the lungs, we also measured levels of TNFα, IL-6 and IL17A in lung tissue, immediately after the Pam3Cys treatments (timepoint 1). Here, we observed no difference between Pam3Cys and control-treated mice for the levels of these pro-inflammatory cytokines in lung tissue (Figure 3e).

Next, we checked whether eosinophilic infiltration in the airways was affected by TLR-2 activation at the time of OVA challenge. Immediately after Pam3Cys treatment, total cell counts showed a tendency to increase in the Pam3Cys-treated group compared to control (Figure 3bc). Interestingly, neutrophils infiltration was significantly increased in Pam3Cys-treated animals (Figure 3c). After the second series of OVA challenges, total cell numbers in BAL were similar between the Pam3Cys-treated and the control group (Figure 4b), but eosinophil numbers were significantly reduced in mice that had received prior Pam3Cys treatment (Figure 4c). Interestingly, analysis of lung tissue cytokine levels revealed increased levels of IL-10, but not of TGFβ in lung tissue at the later timepoint (Figure 4e).

### CD25^+^Foxp3^+^ T Regulatory Cells in the Lung Compartment

**Figure 5:**
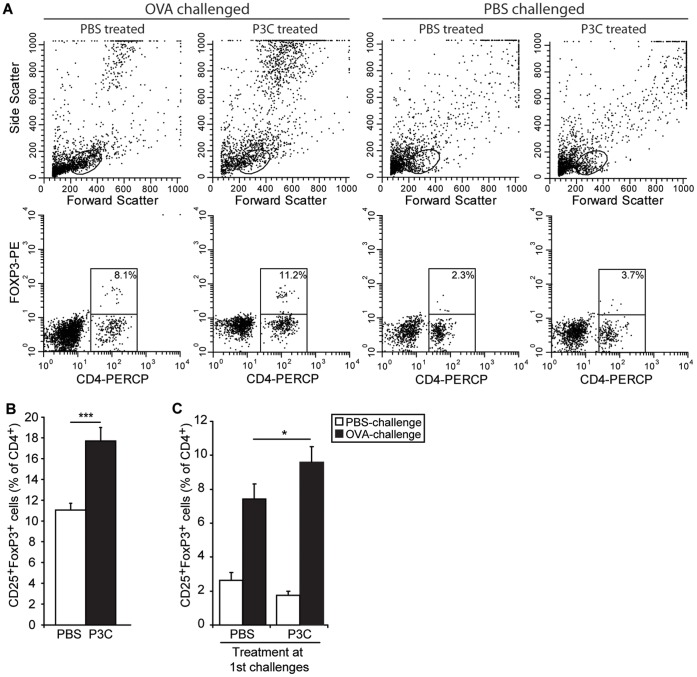
CD25^+^FoxP3^+^ CD4^+^ T cells in BAL. Total cells in the BAL isolated at both timepoints of protocol 2 (Figure 1b) were stained for CD4, CD25 and FoxP3, and analyzed by flowcytometry. A. FACS staining profiles for representative FACS plots of the four groups analyzed at timepoint 2. Forward and Side scatter profiles are used to define lymphocytes, CD4-PerCP/FoxP3-PE was used to identify FoxP3^+^ regulatory T cells. (B, C) Number of CD25^+^FoxP3^+^ cells is shown as a fraction of the total CD4^+^ T cell count. B: the fraction of CD25^+^FoxP3^+^ cells within the CD4^+^ T cells from lung 24 h after the last of three OVA challenges in the presence or absence of Pam3Cys, as indicated. C: the fraction of CD25^+^FoxP3^+^ cells within the CD4^+^ T cells from lung 24 h after the last of three OVA or PBS challenges, three weeks after the first series of challenges as depicted in panel A. Treatment at the first series of challenges is indicated on the X-axis. All values are displayed as average ± SEM. Significance is indicated (*) when p<0.05 or (***) when p<0.005.

To check whether the beneficial effects of Pam3Cys treatment on asthma manifestations could have been mediated by an increased presence of Treg cells, we measured by flow cytometry the numbers of CD4^+^CD25^+^Foxp3^+^ cells in the BAL infiltrates. After the OVA inhalation challenges with Pam3Cys treatment, the proportion of CD25^+^Foxp3^+^ cells in the CD4^+^ population had increased from 11.0±0.7% to 17.7±1.3%, (Figure 5a). Interestingly, this increased presence of Treg cells in the BAL of Pam3Cys treated mice was still observed after the second series of challenge (Figure 5b), although the differences were less pronounced. This indicates that TLR-2 activation at the time of allergen provocation induced the long-term presence of an increased population of regulatory T cells.

## Discussion

Severity of allergic inflammation and AHR in allergic asthma are tightly correlated to the activity of Treg cells, and therapeutic strategies aimed at inducing the expansion of endogenous allergen-specific Treg populations are an attractive future alternative for current treatment options [4], [5]. In this study, we tested whether expansion of Treg cells via TLR-2 activation at the time of allergen challenge can result in long-term protection against asthma manifestations in the mouse. To this end, we treated mice at the time of OVA inhalation challenges with Pam3Cys, a TLR-2 agonist that has been shown to induce Treg cell expansion [6], [17], [19]. We observe a decreased AHR and lung eosinophilia in mice that received prior treatment with the TLR-2 agonist. This effect was initially observed for airway eosinophilia shortly after cessation of Pam3Cys treatment with two different doses of Pam3Cys, whilst the results for AHR showed only a trend towards a reduction in the Pam3Cys treatment groups (protocol 1; Figure 2 and data not shown). However, at prolonged periods after withdrawal of the TLR-2 agonist, lung eosinophilia and AHR were both effectively suppressed upon allergen challenge (protocol 2; Figure 4). Remarkably, AHR was suppressed to the level of PBS-challenged mice at this later timepoint. Since we used an indirect non-invasive measurement of lung function, we cannot draw definite conclusions regarding the airway resistance in our experiments [20]. Nevertheless, we interpret this striking difference in the substitute Penh parameter as a strong indication for an actual difference in lung function between the Pam3Cys-treated group and the control-treated mice.

The effects of Pam3Cys treatment were associated with increased amounts of CD4^+^CD25^+^Foxp3^+^ Treg cells in the BAL at both timepoints, which is in agreement with earlier observations that in contrast to allergen-specific IgE and airway eosiniphilia, AHR is highly sensitive to the presence of lung-resident Foxp3^+^ regulatory T cells [21]. Notwithstanding the induction of this protective response, AHR was exacerbated and lung neutrophilia was induced at the time of TLR-2 activation (Figure 3), indicating that the acute and the long-term effects of Pam3Cys treatment were strongly divergent.

The contrasting results of the acute and long-term effects of TLR-2 activation can be explained by the cells responsible for relaying the Pam3Cys induced signal. Mouse alveolar macrophages, which reside in the alveoli under steady state conditions, express TLR-2 and Pam3Cys treatment induces expression of pro-inflammatry cytokines like IL-6 and TNFα *in vitro* [22]. Airway epithelial cells of the mouse also express TLR-2 [23], and Pam3Cys treatment induced KC expression *in vitro* [24]. Interestingly, activation of TLR-2 in airway epithelial cells has been shown to induce pro-inflammatory cytokine production [25] as well as cleavage of the tight junction protein occludin and the adherens junction protein E-cadherin, allowing transepithelial migration of inflammatory cells [26]. Finally, airway smooth muscle cells will induce IL-8 expression upon TLR-2 activation [27]. Moreover, both eosinophils and neutrophils express TLR-2, and activation of TLR-2 on eosinophils induces the expression of IL-1β, IL-6, IL-8 and GROα, and induces release of eosinophilic cationic protein (ECP) [28]. In dendritic cells, Pam3Cys treatment has been shown to induce IL-6 and IL-10 responses and repress IL-12 expression, thereby favoring Th2 activity [29], [30]. Hence, at the time of the first OVA inhalation challenges in both protocols, TLR-2 agonist could be hypothesized to result in the induction of a pro-inflammatory response by the structural and lung resident cells. Nevertheless, we do not see induction of pro-inflammatory cytokines such as TNFα, IL-6 and IL17A (Figure 3) or suppression of IL-10 or TGFβ (Figure 4) in lung tissue directly after Pam3Cys treatment, indicating that the induction of neutrophilic inflammation does not reflect a generalized pro-inflammatory activation of the airways by Pam3Cys. Clearly, further research is needed to identify the cellular intermediates through which Pam3Cys induces both neutrophilic inflammation and increased AHR. Here, use of adoptive transfer of wild-type BM cells into sublethally irradiated TLR-2-deficient mice might allow identification of the critical cell population for the Pam3Cys-induced effects.

At the same time, Pam3Cys likely acts directly on regulatory T cells,which has been shown to induce expansion and transient loss of suppressive capacity of the Tregs [6], [17], although the TLR-2-induced loss of suppression by Tregs was recently challenged [19]. In our study, the number of Treg cells in the BAL was dramatically increased at the time of Pam3Cys treatment, in line with the reported direct proliferative effect of Pam3Cys on Treg cells (Figure 5). Since we observe exacerbated asthma manifestations at this timepoint, it can be inferred that these TLR-2-expanded Treg cells displayed a reduced suppressive capacity, as shown before [6], [17]. Nevertheless, from our data we cannot exclude that the expanding Tregs have *bona fide* suppressive activity [19], and are merely insufficient in number to functionally repress the TLR-2-induced exacerbated asthma manifestations. We find, however, that the effects of TLR-2 agonist treatment on neutrophilic airway inflammation are lost as soon as 7 days after the last treatment (protocol 1). Remarkably, even after 3 weeks, the expanded Treg subset is retained and has apparently full functionality, resulting in increased levels of lung tissue IL-10, slightly suppressed allergic inflammation and the complete absence of an AHR response upon OVA challenge. Therefore, treatment with Pam3Cys induced a long term protection against AHR, and this effect was associated with the presence of higher numbers of lung Foxp3^+^ Treg cells.

Our findings are in line with previous observations AHR is more sensitive to the presence of lung-resident Foxp3^+^ regulatory T cells than allergen-specific IgE or airway eosinophilia in experimental mouse models of allergic asthma [21]. Nevertheless, studies addressing the role of regulatory T cells in experimental mouse models of asthma have yielded widely divergent results, with some studies observing mainly suppression of AHR [21], some mainly of eosinophils [1], [31], [32], some studies reporting no or little effect at all [33], and finally some reporting strongly strain-dependent effects of Tregs [2]. This discrepancy between studies might well stem from differences in experimental approach, genetic background and treatment regimen used in the various studies. Clearly, further experiments are required to functionally assess the critical requirement for the expanded Treg cell subset on the strong effects on AHR and the relatively mild effects on airway eosinophilia we observe after Pam3Cys treatment.

Studies aiming at the role of TLR-2 signaling in experimental asthma have yielded contrasting results, showing either beneficial or aggravating effects (see ref. [34] for review). These discrepancies are partly due to the fact that various experimental models have been studied, using either TLR-2/−1 or TLR-2/−6 agonists, and using TLR-2 activation during either allergic sensitization or allergen challenge, or even at a distant anatomical location [34]. Importantly, the role of CD4^+^CD25^+^Foxp3^+^ Treg cells was never investigated in these experiments. In our study, we could discriminate the immediate and long-term effects of TLR-2 stimulation, and find that these effects are strongly divergent. This duality in the effects of TLR-2 activation may also explain some of the disparate results previously published concerning the role of TLR-2 in experimental asthma. For instance, Redecke et al. reported an exacerbation of asthma manifestation in mice treated with TLR-2 agonist at the time of subcutaneous sensitization [35]. In contrast, when mice received Pam3Cys intraperitoneally during the challenge phase, asthma manifestations were reduced, which was associated with an IL-12 dependent increase in Th1 activity [36]. The timing and anatomical location of Pam3Cys application are thought to be responsible for the induction of a Th1 response, since these authors also mention an exacerbated asthma phenotype upon intranasal Pam3Cys inoculation. Nevertheless, the observed suppression of asthma manifestations was independent of IL-10 or TGFβ, indicating a lack of involvement of antigen-specific Tregs that can be explained by the distant anatomical location of OVA (intranasal) and Pam3Cys (intraperitoneal) administration [36]. In our study, we specifically show that expansion of lung Treg cells via TLR-2 activation at the time of allergen challenge is associated with long-term protection against asthma manifestations in the mouse. However, our study also has some shortcomings, such as the lack of a significant suppression of AHR in the first Pam3Cys treatment protocol and the lack of histological evaluation of mucus production in all our experiments, which precludes any conclusions to be drawn on this highly relevant asthma parameter.

In conclusion, we show that local TLR-2 activation at the time of allergen challenge results in slightly exacerbated asthma manifestations at the time of treatment, while rendering a long-lasting protection against AHR and allergic inflammation upon allergen provocation at later time points. These data further strengthen the validity of approaches aimed towards *in situ* expansion of regulatory T cell activity as an approach to combat allergic asthma.

## Materials and Methods

### Animals

This study was performed in strict accordance with the national and European regulations for the care and use of laboratory animals. Animal care and use were evaluated and approved of by the Institutional Animal Care and Use Committee of the University of Groningen (IACUC-RuG; permit numbers 5109 and 4253). Specific pathogen-free (according to the Federation of European Laboratory Animal Science Associations) male BALB/c mice (6–8 wk old) were purchased from Charles River (Maastricht, The Netherlands) and housed in macrolon cages in a laminar flow cabinet and provided with food and water *ad libitum*.

### Mouse Models of Asthma

We used two adapted protocols (Figure 1) based on our standard OVA-driven mouse model for experimental allergic asthma [37]. In brief, mice were sensitized intraperitoneally (i.p.) on days 0 and 7 with 10 µg OVA (grade V, Sigma-Aldrich, Zwijndrecht, Netherlands) in 2.25 mg Alum adjuvant (Pierce, Rockford, Illinois). After two weeks, sensitized mice were exposed to four OVA (10 mg/ml in saline) or saline inhalation challenges for 20 min every third day in Protocol 1. In this experiment, mice were treated by intranasal (i.n.) administration of 20 µg Pam3Cys-SKKK (MCA Microcollection GmbH, Germany; endotoxin-free, reconstituted in endotoxin-free water under sterile conditions and stored at –20°C in single-use aliquots) 1 h before the first two of the four OVA inhalation challenges. In protocol 2, mice received 2 series of OVA inhalation challenges, starting 2 weeks after the last i.p. injection. Each series consisted of 3 challenges every third day, and the 2 series were performed at a 3 week interval. Mice were treated by i.n. administration of 20 µg Pam3Cys-SKKK 1 h before the first series of OVA inhalation challenges only, the dose of Pam3Cys-SKKK for intranasal application was based on [38].

### Measurement of Airway Responsiveness in vivo

Twenty-four hours after the last OVA challenge of each series, airway responsiveness was measured in conscious, unrestrained mice using barometric whole-body plethysmography by recording respiratory pressure curves (Buxco, EMKA Technologies, Paris, France) in response to increasing doses of inhaled methacholine (Sigma-Aldrich). Airway responsiveness was expressed in enhanced pause (Penh), as described in detail previously [39].

### OVA-specific IgE ELISA

After measurement of airway responsiveness *in vivo*, mice were anaesthetized and bled by cardiac puncture. Subsequently, serum was collected and stored at –80°C until analysis. Serum levels of OVA-specific IgE were measured by sandwich ELISA as described previously [40]. A reference standard was used with arbitrary units of 1,000 EU/ml. The detection level of the ELISA was 0.5 EU/ml for OVA-specific IgE.

### Differential Cell Counts in the Bronchoalveolar Lavage Fluid

Bronchoalveolar lavage (BAL) was performed immediately after bleeding of the mice by five injections of 1 ml saline (37°C) through a tracheal cannula into the lungs. Cells in the BAL were centrifuged and resuspended in PBS. The total number of cells in the BAL was determined using a Bürker-Türk counting-chamber (Karl Hecht Assistent KG, Sondheim/Röhm, Germany). For differential BAL cell counts, cytospin preparations were prepared (15×g, 5 min, 4°C, Kendro Heraues Instruments, Asheville, North Carolina, US). Next, cells were fixed and stained with Diff-Quick (Dade A.G., Düdingen, Switzerland). Per cytospin, 400 cells were counted and differentiated into mononuclear cells, eosinophils, and neutrophils by standard morphology and staining characteristics.

### Flow Cytometry

For FoxP3 staining, BAL cells permeabilized according to the manufacturer’s instructions (FoxP3 staining kit; eBioscience, San Diego, California, US) and stained with anti-CD4^Per-CP^ (clone RM4-5; Pharmingen BD, Breda, the Netherlands), anti-CD25^APC^ (clone PC61; Pharmingen BD) and anti-Foxp3^PE^ (clone FJK-16; eBioscience). After staining, cells were analyzed with FACScan™ flow cytometer (Becton Dickinson) using CELLQuest™ software.

### Statistical Analysis

All data are expressed as mean ± standard error of mean (SEM). The airway dose-response curves to methacholine were statistically analyzed by a general linear model of repeated measurements (2-way ANOVA) followed by post-hoc comparison between groups. Statistical analysis on BAL cell counts and ELISA were performed using a Student’s t-test (2-tailed, homosedastic). Results were considered statistically significant at the p<0.05 level.
